# Exposure to Environmental Tobacco Smoke in Relation to Behavioral, Emotional, Social and Health Indicators of Slovak School Children

**DOI:** 10.3390/ijerph15071374

**Published:** 2018-06-30

**Authors:** Ludmila Sevcikova, Jana Babjakova, Jana Jurkovicova, Martin Samohyl, Zuzana Stefanikova, Erika Machacova, Diana Vondrova, Etela Janekova, Katarina Hirosova, Alexandra Filova, Michael Weitzman, Lubica Argalasova

**Affiliations:** 1 Institute of Hygiene, Faculty of Medicine, Comenius University, Bratislava, 813 72, Slovakia; ludmila.sevcikova@fmed.uniba.sk (L.S.); jana.babjakova@fmed.uniba.sk (J.B.); jana.jurkovicova@fmed.uniba.sk (J.J.); martin.samohyl@fmed.uniba.sk (M.S.); zuzana.stefanikova@fmed.uniba.sk (Z.S.); diana.vondrova@fmed.uniba.sk (D.V.); janekova@inclinic.sk (E.J.); katarina.hirosova@fmed.uniba.sk (K.H.); alex.filova@gmail.com (A.F.); Michael.Weitzman@NYUMC.ORG (M.W.); 2 Institute of Epidemiology, Faculty of Medicine, Comenius University, Bratislava, 813 72, Slovakia; erika.machacova@fmed.uniba.sk; 3 InClinic s.r.o, Bratislava, 851 01, Slovakia; 4 Department of Pediatrics, New York University, New York, NY, 10016, USA

**Keywords:** Environmental Tobacco Smoke (ETS), Slovak school children, mental health, physical health, Columbia Impairment Scale, Behavioral Problem Index

## Abstract

Environmental tobacco smoke (ETS) exposure has been shown in general as a major environmental risk factor and deserves attention in vulnerable population groups. The aim of the project is to analyze the relationships among the ETS and behavior and health in 6−15-year-old children in Slovakia. The status of physical and mental health of children in relation to exposure to tobacco smoke was examined in a representative group of 1478 school children. The methods used, included anonymous questionnaires filled in by parents, Columbia Impairment Scale (CIS), Behavior Problem Index (BPI) and anthropometry. The prevalence of ETS exposure is the highest in the capital (27%) and southern cities. A significant association was found between ETS and age, socio-economic status, incompleteness of the family, level of mother’s education and a higher prevalence of respiratory diseases (26.7%). The relationships of ETS with emotional (CIS scores ≥ 16) and behavioral functions (BPI score ≥ 14) were significant in children exposed to mother’s or father’s smoking at home. In the multivariate analysis these associations were not significant; the factors such as income and completeness of the family were dominant. The results showed mostly the predominant impact of social factors on the physical and mental health status of Slovak school children.

## 1. Introduction

Numerous national and international studies and health reports provide evidence of the adverse impact of Environmental Tobacco Smoke (ETS) on health during the last decades [1,2,3,4,5,6,7]. ETS, also known as passive cigarette smoke, is linked with premature deaths and many physical and mental disorders [8,9,10,11]. 

The use of the term secondhand smoke (SHS) as a synonym for ETS is not appropriate in light of recent studies. These studies demonstrate that ETS is composed not only of SHS but also of Third Hand Smoke (THS). Third hand smoke is a complex phenomenon resulting from residual tobacco smoke pollutants that adhere to the clothing and hair of smokers and to surfaces, furnishings, and dust in indoor environments. Exposure can even take place long after smoking has ceased, through close contact with smokers and in indoor environments in which tobacco is regularly smoked [12,13,14,15]. Exposure to ETS may cause lung cancer, eye, nose, and throat irritation, may affect the cardiovascular system and may cause stroke [16,17,18,19]. ETS is a serious factor in indoor air pollution, which has an influence on health of predominantly vulnerable population groups—children of all ages [4,9,20,21,22] and pregnant women living in the same house as a habitual cigarette smoker [4,10]. Effects include sudden infant death syndrome (SIDS), asthma, bronchitis and pneumonia, and other respiratory diseases, and an adverse effect on the developing fetus. ETS exposure negatively affects birth outcomes, especially birth weight and behavior disorders [23,24,25]. The associations among ETS exposure during the prenatal or postnatal period and behavioral, emotional problems and mental health in childhood and adolescence have become the subject of many studies [7,26,27,28,29]. Even children whose mothers had prenatal ETS exposure in any one or more of the pregnancy trimesters were more likely to exhibit hyperactivity behaviors as compared with those born to non-exposed mothers [30].

More than a third of all people are regularly exposed to the harmful effects of smoke. This exposure is responsible for about 600,000 deaths per year, and about 1% of the global burden of disease worldwide [31]. In 2004, 40% of children were exposed to ETS in public places worldwide [18]. According to the 2007 Global Youth Tobacco Survey (GYTS), more than 50% of children in Slovakia between the ages of 13–15 were exposed to ETS [32]. 

The aim of the present study is to analyze the relationships among the ETS exposure and emotional and behavioral problems and health in 6–15 year old children in Slovakia. 

## 2. Material and Methods

The status of physical and mental health of children in relation to exposure to tobacco smoke in the family was examined in 1478 school children aged 6–15 years, equal number of boys and girls. The representative group was sampled by random selection of schools in each participating district from regions of the east, middle and western part of Slovakia, of which pupils were randomly selected into Stage I (first to fourth grade) or Stage II (fifth to ninth grade). Of the 2023 questionnaires distributed to the parents of selected grades in 11 primary schools from all over Slovakia 73% (1478) were returned and processed, ranging from 43 to 93% in different schools and localities. Details about the study sample are presented in the previous article of Sevcikova et al. [33]. The study was performed in October and November, 2009. Standard methods (questions based on widely utilized questionnaires) for evaluation of the smoking and socio-economic status of family, environmental conditions, children’s regimens, their health status and anthropometric variables (height, weight) were used. The parents, after informed consent, were given written instructions to fill out the anonymous questionnaires at home and to return it to the teacher via children. This included questions regarding family and child demographic and socioeconomic characteristics, child gender and age, and ETS exposure in the home as determined by whether either the mother or the father reported that they smoked cigarettes at home. Demographic information included family income, ethnicity, maternal and paternal educational attainment, and town/city versus village residence. We set a threshold of 600 € according to the average net money income of private households in Slovakia by the year 2008 [34]. BMI-for-age-gender percentile was used for evaluation of overweight/obesity (the criterion for overweight and obesity was set at the 90th and 97th P of the national standards from 2001) [35]. 

Emotional and behavioral functions of children were assessed using validated questionnaires: The Columbia Impairment Scale (CIS) and The Behavior Problem Index (BPI). CIS is a tool for emotional problems screening by 13 structured questions having fixed response options, with scores ranging from 0 to 52 and tap four major areas of functioning as: (1) interpersonal relations, (2) broad psychopathological domains (e.g., anxiety, depression or behavior problems); (3) functioning in job or school and (4) use of leisure time. When an item is not applicable (e.g., problems getting along with siblings for an only child), or missing, the mean of all scored items for that individual was assigned as that item score. A score of 16 or higher is considered clinically impaired [36,37,38]. The BPI has 25 questions with scores ranging from 0 to 25 (how much of a problem with antisocial behavior, cheating, disobedience at home, at school, bullying or cruel behavior, anxious/depressed behavior, headstrongness, short temper, irritability, sensitivity, nervousness, hyperactivity, difficulty concentrating, impulsive thinking, restlessness, conflicting behavior or social isolation, social immaturity). A score of 14 or above indicates behavior problems [33,39].

Surveys were included if they were missing fewer than five responses. For included surveys with missing data, the data was imputed by replacing the missing values from blank responses with the average of all the other questions.

The association between maternal and paternal smoking in the household and emotional and behavioral problems (CIS ≥ 16) and (BPI ≥ 14), and other child, maternal, and family characteristics were examined in bivariate analyses using chi-square tests and crude odds ratios with 95% confidence intervals. The age (6–10 or 11–15 years), gender, residence (town or country), income (≤600 € or >600 €) were investigated as well as basic life style habits and anthropometric characteristics (BMI) in relation to ETS exposure in the household. The most important questions on CIS and BPI were analyzed separately in bivariate analysis with household smoking. Multivariate analyses were performed to identify those factors independently associated with increased scores, indicative of child emotional and behavioral problems, for all of these variables using adjusted odds ratios. All analyses were conducted in SAS (SAS Institute, Cary, NC, USA and SPSS 25 (International Business Machines Corp.; New Orchard Road; Armonk, New York, USA) programs. The statistically significant level was determined at *p* values < 0.05.

The project was approved by Ethical Committee Faculty of Medicine, Comenius University Bratislava, Slovakia and by Institutional Review Board of New York University School of Medicine, New York, U.S.A (IRB number: 09-0331).

## 3. Results

The prevalence of ETS in Slovak children (mother or father reported smoking at home) was 19% (Table 1). It was significantly the highest in the capital (27.6%) and southern border cities (24–27%) [33]. The characteristics of smoking with social status of families are presented in Table 1. 

Significant relationships between ETS and the level of mother’s and father’s education, father’s employment, socio-economic status and completeness of the family were found in bivariate analysis (Table 2). The older school children were more exposed to ETS, but not significantly. Exposure to ETS decreased with the level of parental education—especially the mother. The relationships with socio-economic status and incompleteness of the family have been shown, as well as the marginally significant higher frequency of respiratory system diseases (bronchitis, pneumonia) during the last year in children exposed to ETS (*p* = 0.04). Children exposed to ETS have worse eating habits and regimen, watch TV and play games longer during the day. The relationship between ETS and lower physical activity and sports was statistically significant. The exposure to ETS also was associated with a significantly increased prevalence of overweight/obesity in this sample of Slovak children (*p* = 0.01) (Table 2). 

There were 4.6% boys and 2.5% girls in our sample who were active smokers. These children were exposed to ETS in the family (boys—88%, girls—94%). They were excluded from further analyses with the final sample being 1373 non-smoking children.

The relationships between environmental tobacco smoke exposure (father or mother smoking at home) with disorders of emotional and behavioral functions (CIS scores ≥ 16 and BPI scores ≥ 14) were significant in bivariate analysis (ORcrude, CIS ≥ 16 = 1.76 (95% CI = 1.00–3.14; ORcrude, BPI ≥ 14) = 1.66 (95% CI = 1.07–2.60) (Table 3).

The association of a smoking mother (at home or elsewhere) with child emotional problems (CIS) was significant as demonstrated in Table 4. 

In the analysis of individual behavioral and emotional characteristics of the child in relation to mother’s smoking the association with antisocial behavior of children, anxiety, depressed mood were found to be statistically significant in bivariate analysis (Table 5). The importance of the mother’s influence on some behavioral characteristics (BPI) of school children has been shown. The association of mother’s smoking with individual emotional characteristics of the child (CIS) was not significant.

In contrast, social factors (income and completeness of the family) were statistically significant in multivariate analyses among factors independently associated with children’s behavioral (BPI) and emotional (CIS) problems. The exposure to ETS at home (mother or father smoking at home) was not significant in relation to disorders of emotional functions (CIS) and behavioral functions (BPI) in multivariate analysis (Table 6 and Table 7). 

## 4. Discussion

The analysis of the exposure of Slovak school children to ETS revealed a high number of smoking parents (21% of mothers and 37% of fathers). There were 8% of ex-smokers among mothers and 12% among fathers. The results showed that 19% of children are exposed to ETS in the household. The significant differences among regions were revealed with the highest numbers of exposed children in the capital (Bratislava) and the cities in the south of Slovakia.

The first data on household ETS exposure of Slovak school children are from 1996 in the framework of the Central European Study on Air Pollution and Respiratory Health (CESAR) study on a sample of 2531 7–11 year old children. That study demonstrated the very high prevalence of ETS exposure—48.4% children [40]. 

Slovakia belongs to the countries with a relatively high prevalence of smoking. The WHO Report on the Global Tobacco Epidemic presented the age and sex standardized adult daily smoking prevalence in 2009 in Slovakia as 29% (38% men and 19% women) [6]. Comparable national data of Tobacco and Health Education Survey in 2014 showed the same prevalence 29%, but with the higher percentage in women—24% and lower in men—34% [41]. The prevalence of smoking parents in our study was 21% of smoking mothers and 37% of smoking fathers. 

The home is the primary source of ETS exposure for infants and children and a major source of ETS exposure for non-smoking adults, mostly mothers [10,42,43,44]. In our study the older children were more exposed to ETS, however not significantly. Children of parents with lower education (especially mothers) are significantly more exposed to ETS. Many prior studies have found lower maternal education and poverty to be associated with child ETS exposure [45,46,47,48]. 

Children exposed to ETS were significantly more often ill, e.g., for respiratory system diseases. Many studies have reported adverse effects of prenatal and/or postnatal exposure on children’s respiratory health [18,49]. Among children whose mothers smoke, the risk of asthma is higher. A child with asthma is twice as likely to end up in the hospital if family members smoke [50]. Children exposed to ETS have an increased risk of developing lung cancer in adulthood, even if they never smoked [51]. These serious consequences from exposure to tobacco smoke for children’s respiratory health urgently needs to be reduced.

Children whose parents smoke and children from low-SES families are still most likely to be exposed to tobacco smoke [52]. The known relationships of ETS exposure with socio-economic status and incompleteness of the family [6,18] also have been confirmed in our study. 

Children exposed to ETS have worse living habits, worse nutrition, less sport activities, and watch more TV and are more likely to play computer games. Results demonstrate more sedentary daily activities in the exposed children. These negative habits in exposed children may be influenced also by lower socioeconomic status or education of a smoking mother [53,54].

Slovak school children exposed to ETS tend to be overweight and obese similar to those in the US [55]. 

The significant association between ETS exposure and mental health has been found in many studies [38,56]. Numerous potential pathways have been proposed, including alterations in brain functions or structure due to exposure to nicotine or to the other chemicals in exhaled tobacco smoke [29]. The potential social, genetic and behavioral confounders (e.g., psychological stress, maternal depression, and family functioning), as well as potential causal neurobiological pathways (e.g., dopamine system) remain as potential contributors [11]. 

Children living with smokers are at increased risk for emotional or behavioral problems [38]. The associations among developmental, neurocognitive or behavioral problems in children and tobacco exposure may have substantial effects on functioning and quality of life and also other risks, in which genetic and societal factors have an essential impact [55,57,58,59,60]. Mental and physical load in school children could be associated also with the educational process [61], that we did not study in this sample. The exposure to environmental tobacco smoke, even at extremely low levels, is associated with decreases in certain cognitive skills in children and adolescents [62]. Both animal model and human epidemiologic data clearly suggest a causal relationship between prenatal tobacco exposure and adverse behavioral and neurocognitive effects on children [63]. Many studies described the association between prenatal tobacco or postnatal ETS exposure and the following adverse behavioral and emotional outcomes: conduct disorder, attention-deficit/hyperactivity disorder, poor academic achievement, and cognitive impairment [59,63,64,65].

Children exposed to ETS in the present study scored higher (worse) in emotional and behavioral problems (score CIS and BPI) only in bivariate analysis. The more bivariate analysis of individual behavioral and emotional characteristics performed in this study also revealed associations with ETS exposure in the households. However, the exposure to ETS was not significant in relation to disorders of emotional functions (CIS scores ≥ 16) and behavioral functions (BPI score ≥ 14) in multivariate analysis in the current as well as in the previous study [33]. The social factors (income and completeness of the family) were dominant, eliminating the statistical significance found in bivariate analysis. Further neurobiological research to establish causal pathways is needed [11,66].

Results of this study also confirm that smoking of parents or other adult members of the family are significantly associated with active smoking in children. There were 4.6% boys and 2.5% girls in our sample with smoking experiences and they were exposed to passive smoking in the family as well (boys–88%, girls–94%). The causal relationship between environmental tobacco exposure and adverse behavioral and cognitive outcomes is suggested in many studies [55,59,64].

Children living in incomplete families or families earning less than the average income had increased rates of behavioral and emotional problems in multivariate analyses. This analysis also confirmed the relation of emotional problems with the age of children which may be related to puberty and adolescence. The number of children exposed to household smoking significantly differs in different Slovak regions; the highest prevalence was in the capital and the south of Slovakia. These areas need particular attention to anti-smoking interventions.

Our results confirm that, children exposed to ETS tend to be from lower socioeconomic families. Negative attitude toward smoking alongside knowledge of the effects of ETS are very important in interventions for decisions to maintain a smoke-free home [67]. 

The strength of this study is the representative sample of Slovak school children. Standardized and internationally accepted measures also were used (BPI, CIS—behavioral and emotional functions). Voluntary participation may represent some limitation by omission of “smoking” families. More accurate estimate of ETS exposure in children may have resulted if the evaluation of a biomarker were used. Cotinine levels in urine may provide tangible and objective evidence for ETS [68]. The effects of other potential environmental toxins on children also have not been investigated. Biomarkers of tobacco exposure help to clarify the role tobacco chemicals play in influencing health both in childhood and beyond [69]. 

The results showed mostly the predominant impact of social factors on health status and health behaviors of Slovak school children lowering the apparent impact of ETS. Nevertheless, ETS is an important public health problem in Slovakia and should lead to further research on ETS interactions with social factors on physical and mental health of children and parents as well. They are important for application in clinical practice and public policy. 

## 5. Conclusions

The results showed the predominant role of social factors on health status and health behaviors of Slovak school children, eliminating the association of ETS with these domains found in crude (bivariate) analysis. Nevertheless, ETS is an important public health problem in Slovakia and should lead to further research on ETS interactions with social factors on physical and mental health of children and parents as well.

The exposure to ETS has been documented as a risk factor in children with lower education of parents (especially mothers), lower socio-economic status of the family and incompleteness of the family. 

The significant relationship between negative dietary habits, lower exercise, longer TV watching, overweight and the ETS exposure has been confirmed as in other studies. Our study revealed a lack of intervention in the area of the children’s protection from exposure to toxic tobacco smoke. In Slovakia more public health activities in children protection from tobacco smoke in the family and household smoking bans are urgently needed. It is necessary to continue systematically and more vigorously in activities that lead to secure children’s rights to protection against smoking, which started in Slovakia in the year 2000. Education concentrated on family and society and systematic preventive health-care during childhood and adolescence belong to fundamental aspects of public health policy. An important role is played by the education of all population groups and an intensive campaign against the risk to children’s health caused by ETS exposure.

## Figures and Tables

**Table 1:** Characteristics of the sample of Slovak children (*n* = 1478).

Indicator *	*n*	%
**Age group (years)**		
6–10	953	64.8
11–15	517	35.2
**Gender**		
Boys	738	50.6
Girls	720	49.4
**Mother education**		
Primary	51	3.5
High school	360	24.6
High school completed	739	50.6
University	311	21.3
**Mother smokes**		
Yes	309	21.3
No (ex-smoker)	114	7.9
No	1025	70.8
**Father education**		
Primary	41	2.9
High school	566	40.4
High school completed	505	36.1
University	289	20.6
**Father smokes**		
Yes	484	36.5
No (ex-smoker)	161	12.2
No	680	51.3
**The child smokes**		
Yes, more times	22	1.54
Yes, one time	30	2.11
No, never	1373	96.35
**Mother or father smokes**		
Yes	610	41.16
No	872	58.84
**Mother or father smokes at home**		
Yes	278	18.8
No	1204	81.2
**The child lives in the**		
Uncomplete family	287	19.62
Complete family	1176	80.38
**Monthly household income**		
≤400 €	146	11.6
401–600 €	233	18.6
> 600 €	877	69.8
**Number of siblings**		
0	289	20.2
1–2	949	66.3
≥3	194	13.5
**Residence**		
Urban	1169	81.01
Rural	274	18.99

* There are some data missing in each variable category.

**Table 2:** Relations between selected indicators and children’s exposure to ETS (*n* = 1373) *.

Indicator **	Exposure ETS −	Exposure ETS +	*p*-Value
	(%)	(%)	
**Age (years)**			0.08
6–10	82.3	17.7	
11–15	78.3	21.7	
**Mother education**			<0.0001 ***
Primary	51.2	48.8	
High school	73.5	26.5	
High school completed	82.5	17.5	
University	89.7	10.3	
**Father education**			<0.0001 ***
Primary	52.6	47.4	
High school	75.3	24.7	
High school completed	83.3	16.7	
University	92.5	7.5	
**Father employment**			<0.0001 ***
Unemployed	54.5	45.5	
Employed	81.7	18.3	
**Monthly household income**			<0.0001 ***
≤400 €	60.9	39.1	
401–600 €	70.2	29.8	
>600 €	85.4	14.6	
**Completeness of the family**			<0.0001 ***
No	69	31	
Yes	83.7	16.3	
**Respiratory system diseases**			=0.04 *
No	81.7	18.3	
Yes	73.6	26.4	
**Child eating healthy (vegetable, fruits, number of portions)**			=0.03 *
yes	82.6	17.4	
no	77.8	21.1	
**Child doing sports**			0.04 *
No sports, rarely	72.9	27.7	
Recreationally, 1–2 times a week	80.8	19.2	
Regularly more time a week/daily	82.5	17.5	
**TV/Games combined**			<0.0001 ***
≤2 h daily	84.3	15.7	
>2 h daily	66.1	33.9	
**Overweight/Obesity**			=0.01 *
No	82.3	17.7	
Yes	75.58	24.42	

* ETS = mother or father smokes at home; ** There are some data missing in each variable category; * *p* < 0.05; *** *p* < 0.001.

**Table 3:** Relations between behavioral functions (BPI) and emotional functions (CIS) and the exposure of children to father’s or mother’s smoking at home (*n* = 1373) (bivariate analysis).

Indicator	Exposure ETS (%)	Risk Estimation			
		OR (Crude)	95% CI	Chi-Square	*p*-Value
**CIS score ≥ 16**	6.67	1.76	1.00–3.14	4.37	0.05 *
**BPI score ≥ 14**	11.72	1.66	1.07–2.60	5.19	0.02 *

* *p* < 0.05.

**Table 4:** Relations between emotional functions (CSI) and behavioral functions (BPI) and the exposure of children to mother’s smoking ^†^ (*n* = 1373) (bivariate analysis).

Indicator	Exposure ETS (%)	Risk Estimation			
		OR (Crude)	95% CI	Chi-Square	*p*-Value
**CIS score ≥ 16**	8.05	2.13	1.10–4.11	5.30	0.02 *
**BPI score ≥ 14**	11.72	1.53	0.89–2.66	2.37	0.12

* *p* < 0.05; ^†^ mother smokes elsewhere.

**Table 5:** Behavioral characteristics of children (BPI) and mother’s smoking ^†^ (*n* = 1373) (bivariate analysis).

Behavioral Characteristics of Children	Exposure ETS (%)	Risk Estimation			
		OR (Crude)	95% CI	Chi-Square	*p*-Value
**Antisocial behavior of children**					
She/he cheats or tells lies	33.69	1.31	1.00–1.74	3.57	0.05 *
She/he is disobedient at school	22.97	1.50	1.07–2.05	5.90	0.015 *
She/he hangs around with kids who get into trouble	21.43	1.27	0.92–1.75	2.02	0.15
**Anxiety and depressed mood**					
She/he has sudden changes in mood or feelings	25.00	1.27	0.93–1.72	2.27	0.13
She/he feels or complains that no one loves him/her	22.26	1.67	1.20–2.31	9.36	0.002 *

* *p* < 0.05; ^†^ mother smokes elsewhere.

**Table 6:** Factors associated with Children’s Behavioral Problems (BPI ≥ 14) among Children in Multivariate Analyses (*n* = 1373) ^†^.

	Adjusted OR	95% CI
**ETS**		
Yes	1.0245	0.58–1.81
No	1	−
**Age**		
6–10 years	1	−
11–15 years	0.73	0.43–1.24
**Gender**		
Male	1	−
Female	0.60	0.37–0.96
**Residence**		
Urban	1	−
Rural	1.57	0.89–2.75
**Maternal education**		
Primary + Some high school	1.08	0.62–1.87
High school diploma + University	1	−
**Paternal education**		
Primary + Some high school	1.38	0.83–2.29
High school diploma + University	1	−
**Income**		
≤600 €	1.95	1.11–3.44 *
> 600 €	1	−
**Completeness of the family**		
No	1.91	1.02–3.55 *
Yes	1	−

^†^ There are some missing data in each variable category; * *p* < 0.05.

**Table 7:** Factors associated with Children’s Emotional Problems (CSI≥16) among Children in Multivariate Analyses (*n* = 1373) ^†^.

	Adjusted OR	95% CI
**ETS**		
Yes	1.25	0.57–2.72
No	1	−
**Age**		
6–10 years	1	−
11–15 years	2.05	1.06–3.94 *
**Gender**		
Male	1	−
Female	0.64	0.35–1.26
**Residence**		
Urban	1	−
Rural	1.44	0.65–3.18
**Maternal education**		
Primary + Some high school	0.54	0.22–1.32
High school diploma + University	1	
**Paternal education**		
Primary + Some high school	0.51	0.24–1.07
High school diploma + University	1	−
**Income**		
≤600 €	2.31	0.93–4.75 *
>600 €	1	−
**Completeness of the family**		
No	3.54	1.58–7.94 **
Yes	1	−

^†^ There are some missing data in each variable category; * *p* < 0.05; ** *p* < 0.01.
